# Oxidative modification and electrochemical inactivation of *Escherichia coli* upon cold atmospheric pressure plasma exposure

**DOI:** 10.1371/journal.pone.0173618

**Published:** 2017-03-30

**Authors:** Marlène Dezest, Anne-Laure Bulteau, Damien Quinton, Laurent Chavatte, Mickael Le Bechec, Jean Pierre Cambus, Stéphane Arbault, Anne Nègre-Salvayre, Franck Clément, Sarah Cousty

**Affiliations:** 1 UMR 5254, IPREM, Université de Pau et des pays de l’Adour, Pau, France; 2 NSysA group, ENSCBP, CNRS UMR 5255, ISM, Université de Bordeaux, Pessac, France; 3 INSERM UMR-1048 I2MC, Université Paul Sabatier, Toulouse, France; 4 Faculté de Chirurgie Dentaire de Toulouse, centre Hospitalier Universitaire de Toulouse, Université Paul Sabatier, Toulouse, France; Defence Research and Development Organisation, INDIA

## Abstract

Cold atmospheric pressure plasmas (CAPPs) are known to have bactericidal effects but the mechanism of their interaction with microorganisms remains poorly understood. In this study the bacteria *Escherichia coli* were used as a model and were exposed to CAPPs. Different gas compositions, helium with or without adjunctions of nitrogen or oxygen, were used. Our results indicated that CAPP induced bacterial death at decontamination levels depend on the duration, post-treatment storage and the gas mixture composition used for the treatment. The plasma containing O_2_ in the feeding gas was the most aggressive and showed faster bactericidal effects. Structural modifications of treated bacteria were observed, especially significant was membrane leakage and morphological changes. Oxidative stress caused by plasma treatment led to significant damage of *E*. *coli*. Biochemical analyses of bacterial macromolecules indicated massive intracellular protein oxidation. However, reactive oxygen and nitrogen species (RONS) are not the only actors involved in *E*. *coli’s* death, electrical field and charged particles could play a significant role especially for He-O_2_ CAPP.

## Introduction

Cold Atmospheric Pressure Plasmas (CAPPs) are partially ionized gases generated at near ambient temperature and pressure. Depending on specific experimental conditions, a non-equilibrium state is achieved which keeps the plasma close to room temperature with production of a reactive mix containing electrons, positive and negative ions, groups of reactive species such as reactive oxygen (ROS) or nitrogen (RNS) species, neutral species in fundamental and excited states (metastables and radiative states) and highly energetic photons [1–3]. There is no universal CAPP, the composition as well as the ratio between the different energetic components depends on the set up employed and the gas mixture used to generate the plasma [3]. The number of potential applications of CAPPs in Biology and Medicine has increased significantly in the last few years leading to the emergence a of a new scientific field called Plasma Medicine [4]. As part of this evolving field, numerous studies have reported promising results regarding the use of CAPPs as a potential for microbial inactivation, treatment of chronic infections [5], wound healing [6] and medical instrument decontamination [7]. This process has numerous advantages particularly for the sterilization of temperature sensitive material and promotes an efficient inactivation of many different types of microorganisms such as phages and viruses [8, 9], bacteria [10, 11], spores [12], fungi [13] and parasites, even those which are resistant to more conventional methods [14]. The exact mechanisms of the interactions between CAPPs and microorganisms remain unclear but three pathways have been proposed: (1) direct permeabilization of the cell membrane or wall leading to leakage of cellular components; (2) critical damage of intracellular proteins by Reactive Oxygen or Nitrogen Species (RONS); and (3) direct DNA damage [15, 16]. The main factors suggested to provide bactericidal effects of CAPPs, are, RONS, charged particles, ultraviolet radiation and electrical field [17]. Many studies have been conducted to identify the role of each element in the processes of bacteria inactivation [18–20].

It is clear that the choice of a plasma-generating device and its operating conditions influence antimicrobial outcomes, since germicidal effects can occur immediately or several hours post-treatment [10, 21, 22]. The addition of oxygen or nitrogen to the feed gas may enhance the treatment efficiency [23, 24]. Microbiological samples can be treated under various conditions depending on the type of microorganism [25]. When they are in suspension, CAPPs mostly interact with the liquid, creating new species by the impact of the plasma on the liquid surface and the interaction with the sample. After CAPP exposure, studies have usually revealed the presence of NO_2_^-^, NO_3_^-^, and H_2_O_2_ in the liquid media sometimes at very high concentrations [26–29], which may or may not be associated with a pH change [26, 27].

The aim of this study was to focus on the interactions between active species produced by 3 different types of plasma: He, He/O_2_ and He/N_2_, and the bacteria *E*. *coli* and begin to assess how the chemistry involved in the liquid and the electrical field associated with the guided ionization wave or generated in the environment of the CAPP or at the bacterial membrane may lead to bacteria inactivation. Our results demonstrated that plasma treatment (He or He/N_2_) induced a significant inactivation of *E*. *coli*. This is accompanied by selective carbonylation of proteins with no formation of lipid peroxidation, or protein nitration. However the effects of He/O_2_ plasma treatment, which is the most aggressive, led to the conclusion that probably the involved chemistry in the liquid could not, completely explain the observed bacteria inactivation.

## Materials and methods

### Helium Guided Ionization Wave (He-GIW) device

The plasma process consists of the production of guided ionization waves at atmospheric pressure and room temperature. It has been previously characterized in other studies [30–34]. Briefly, the reactor is a dielectric alumina tube (internal diameter, Øinternal = 1.14 mm and external diameter, Øexternal = 2.5 mm) into which a tungsten filament (Ø = 125 μm) is inserted and powered at a high-voltage. A metallic cylinder is fixed around the dielectric tube and grounded, thus allowing the application of high voltage between the tungsten filament and the metallic cylinder. This configuration limits the current and avoids the formation of electrical arcs. Due to the very thin diameter of the tungsten filament, a point effect induces the formation of specific ionization waves, which are guided by the dielectric tube and propagated in the surrounding air for up to several centimeters. Process gas was either He, He /1% N_2_ mixture or He /1% O_2_ mixture at a 2 standard liters per minute (slm) flow rate. Plasma was generated by applying a 5.5 kV, 10 kHz, 1% duty cycle, positive microsecond pulsed wave potential between the two electrodes [30, 31].

### Chemicals and antibodies

All chemicals were purchased from Sigma-Aldrich (Saint Quentin Falavier, France). Phosphate buffered saline, PBS (1.5 mM KH_2_PO_4_, 155 mM NaCl, 2.70 mM Na_2_HPO_4_, 7H_2_O, pH 7.2) was used in this study.

### Bacteria and culture conditions

*Escherichia coli* CIP 53126 provided by Dr Sarah Cousty (Université de Toulouse, France) was used for all experiments. *E*. *coli* was grown aerobically overnight at 37°C in Luria-Bertani (LB) medium. In order to have bacteria in the exponential phase, a fraction of the culture was suspended in fresh medium a few hours before the experiments. The optical density (600 nm) was measured and bacteria were separated from their nutritive medium by centrifugation. Then the pellet was washed twice with phosphate buffered saline (PBS). The supernatant was replaced by fresh PBS to give a suspension of 10^6^ bacteria/ml.

### Plasma treatment

Three different plasma mixtures were used: He, He-1%O_2_ and He-1%N_2_. Plasma was turned on 15 minutes before sample treatment to allow its stabilization. Similarly, a two minute wait was required between each change of plasma mixture. For each condition, 1ml of the bacterial suspension at 10^6^ bacteria/ml was deposited in a 12 well plate. The distance between the sample surface and the output of the reactor was fixed at 15 mm. Samples were exposed to different exposure durations from 1 to 10 min. Immediately after exposure, treated samples were placed at 4°C to prevent bacteria proliferation.

### Inactivation of *E*. *coli* after plasma exposure

Three complementary methods were used to follow the effect of plasma treatment on the growth and viability of *E*. *coli*. ***Colony forming unit (CFU) counting*** was used to quantify the effect of plasma treatment. Immediately, 1 hour to 24 hours after the end of the plasma treatment, samples were diluted (1: 1000) in PBS and 100 μl of the dilution were spread on agar in Petri dishes. After, 24 hr incubation at 37°C the percentage of surviving cells was calculated by counting CFUs. Note that a control sample, with He gas (with the power supply turned off), was processed every time. ***The most probable number (MPN) method*** was used to measure the bacterial survival, a liquid method that prevents them from experiencing spreading stress on the Petri dishes and to reduce the volume of waste. This liquid phase method on a microplate is based on the limits of dilutions using a statistical count [35].

***BD Cell Viability Kit*** was useful to ensure that bacteria were dead and not in a viable but non-culturable (VBNC) form. Live cells have intact membranes and are impermeable to dyes such as propidium iodide (PI), which only leaks into cells with compromised membranes. Thiazole orange (TO) is a permeant dye and enters all cells, depending on their membrane potential. Thus a combination of these two dyes provides a rapid and reliable method for discriminating live and dead bacteria. An intermediate or injured population (cell with no total membrane disruption) can often be observed between the live and dead populations. It was performed as described by the manufacturer (Thermofischer, Saint Aubin, France). Briefly, bacteria were stained using the BD Cell Viability Kit and the fluorescence is monitored on a BD Accuri^™^ C6 flow cytometer (BD Biosciences, Le Pont de Claix, France) for 30 seconds at the fast flow rate (66 μL/min) with the SSC-H threshold at 10,000 to exclude debris.

### Treatment induced morphological changes in treated bacteria

Scanning electron microscopy (SEM) (Trigenotoul, Toulouse, France) was used to investigate the bacterial structural and morphological changes after 10 min plasma treatment and 2 hr storage. Bacteria were centrifuged and pellets were fixed in 2% glutaraldehyde. Samples were then kept at 4°C before analysis.

### Plasma Activated Liquid (PAL) effect on bacteria

PBS was treated for 10 min with He, He-1%N_2_ or He-1%O_2_ plasma. Immediately or after being placed at 4°C for 2 hr, treated liquid was incubated with bacteria. The effect of PAL on *E*. *coli* was evaluated using CFU counting as described in a previous section.

### Detection and identification of the main species produced in PBS after He and He-N_2_ plasma treatment

The nature and the concentrations of stable chemical species produced in PBS after plasma treatment, were measured using an electrochemical method named cyclic voltammetry at platinized microelectrodes [36, 37]. In our experiments, only hydrogen peroxide and nitrite are chemically stable enough to be monitored. PBS was treated with each of the two plasmas for different times as previously described and analyzed immediately at the end of the plasma exposure. The kinetics of the species produced in PBS was characterized depending on treatment duration and gas mixture used.

### Determination of hydrogen peroxide and nitrite

Hydrogen peroxide levels generated by He-O_2_ plasma treatment were measured using the Amplex Red assay according to the manufacturer (Thermofischer, Saint Aubin, France). Calibration was performed in PBS using hydrogen peroxide concentrations varying from 5 to 100 μM. Levels of nitrite NO_2_^-^ were determined using the Griess assay according to the manufacturer’s protocol (Thermofischer, Saint Aubin, France).

### Mimics

To determine if the chemical species detected by cyclic voltammetry are those involved in bactericidal effect of PAL, untreated PBS was supplemented with H_2_O_2_ and NO_2_^-^ at the concentrations measured in PAL after 10 min He-plasma treatment. This solution was then incubated for 2 hr with bacteria. The percentage of surviving cell was calculated by counting CFUs.

### Detection of carbonylated and nitrated proteins

Following plasma treatment, *E*. *coli* bacteria were centrifuged, suspended in PBS and sonicated three times for 5 seconds. After 10 min of centrifugation at 14,000 g, the supernatant was recovered. Total protein concentration was evaluated using the DC protein assay kit (Biorad, Marnes la Coquette, France). Carbonylated proteins were detected and analyzed after the derivatization of protein carbonyl groups with 2,4-dinitrophenylhydrazine (DNPH) (Protein Oxidation Detection Kit, OxyBlot^™^, Millipore, Molsheim, France). Derivatized samples were resolved by SDS-PAGE in a 4–20% acrylamide gel and electrotransferred onto a Hybond nitrocellulose membrane (GE Healthcare, Piscataway, NJ, USA). The primary antibody for Western blotting was raised against dinitrophenylhydrazone, and primary antibody binding was detected with a peroxidase-conjugated secondary antibody and a chemiluminescent substrate (ECL plus Western blotting detection system, GE Healthcare). Western blots were quantified using Image J. Anti-3nitrotyrosine was used to detect nitrated proteins (Abcam, Paris, France).

### Lipid A and HNE quantification

Lipid A quantification and HNE-modified protein by ELISA were performed by dot blot as previously described [38].

### Statistical analysis

Results were expressed as mean ± SEM and analyzed using GraphPad Prism 5 Software. The Mann—Whitney and one-way ANOVA tests were used to compare data sets. Statistical significance was set at *P* < 0.05.

## Results and discussion

### Effect of treatment time and gas composition on bacteria inactivation

**Fig 1:**
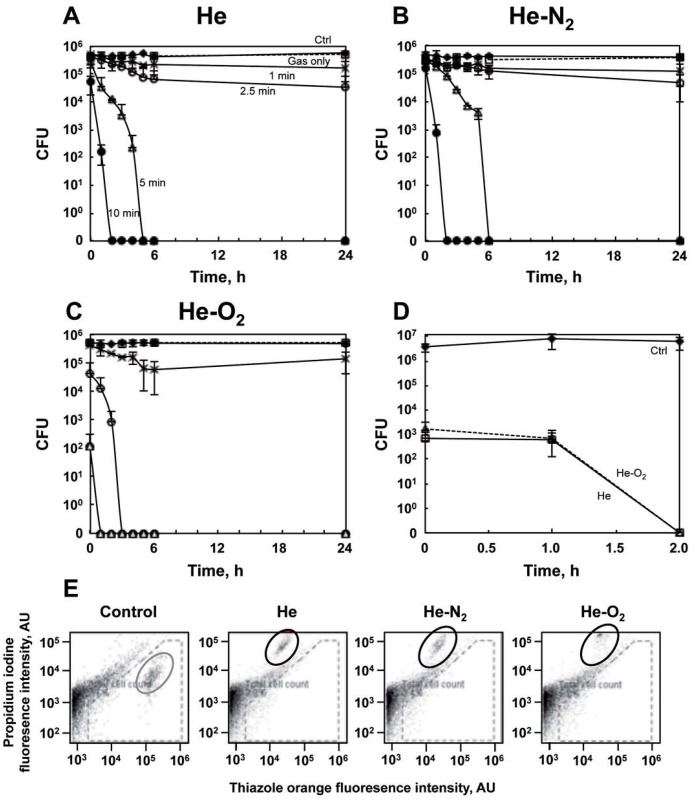
Effect of plasma exposure on bacteria inactivation and viability. A: Survival curves obtained by CFU counting method for *E*. *coli* bacteria exposed to He plasma for different times (black diamond, control; white square, He gas only; black cross, 1 min He plasma treatment; white circle, 2 min 30 s; white triangle, 5 min and black circle, 10 min) and left for different post-treatment storage times 2, 3, 4, 5, 6 and 24 h in PBS at 4°C. B: Survival curves for *E*. *coli* exposed to He-N_2_ plasma for different times (black diamond, control; white square, He only; black cross 1 min He-N_2_ plasma treatment; white circle, 2 min 30 s; white triangle, 5 min and black circle, 10 min); and left for different post-treatment storage times. C: Survival curves for *E*. *coli* exposed to He-O_2_ plasma for different times (black diamond, control; white square, He only; black cross, 1 min He-O_2_ plasma treatment; white circle, 2 min 30 s; white triangle, 5 min and black circle, 10 min) and left for different post-treatment storage times. D: Survival curves obtained by MPN method for *E*. *coli* exposed to He plasma (white square) and He-O_2_ plasma (white triangle) for 10 min and different post-treatment storage times (1 hr and 2 hr). Non-treated bacteria (black diamond). The values are means ± SEM of 3 separate experiments. E: Flow cytometry analysis of bacteria after 10 min plasma treatment (He, He-O_2_ and He-N_2_) and one hour post-treatment storage. Cells were initially gated on an FL2-A vs SSC-A plot (dashes). Simultaneous staining with thiazole orange (TO) and propidium iodide (PI) allowed the distinction between live (TO^+^PI^−^, red circle), dead (TO^+^PI^+^, black circle), and injured (TO^+^PI^int^, dark grey) cell populations, revealing increased cell injury and death in the treated sample as expected. The TO^−^PI^+^ population was excluded from the analysis as debris.

In order to maximize the effects of the treatment on *E*.*coli*, plasmas with different energetic components and different RONS levels were required. Three plasmas were used in this study using different gas mixtures (helium alone or with 1% oxygen or 1% nitrogen). We used a classical colony counting method to determine antibacterial efficacy after plasma treatment. Fig 1A represents number of surviving bacteria measured during 24hr after He plasma treatment. As expected, the bacterial CFU counting shows that treatment with gas only (discharge turn off) does not induce cell death (Fig 1A). For He and He-N_2_ plasmas, treatment durations below 5 min do not induce significant inactivation of bacteria (Fig 1A and 1B). However, treatment for 5 min induced total inactivation of the bacterial population if post-treatment storage of the bacteria was more than 4 hours, indicating that inactivation was related to both treatment and post-treatment storage time. The results obtained with He-O_2_ plasma are different from those obtained with the two others (Fig 1C). Even a short exposure of 2 min 30 sec is sufficient to inactivate the whole population after 3 hours incubation. One hour post-treatment storage after 5 min plasma exposure time led to undetectable levels of bacteria populations whereas it took about 5 hours and 2 hours post-treatment storage respectively to have the same effects with other He and He-N_2_ plasmas (Fig 1A and 1B).

To assess the decontamination efficacy of plasma treatment we used the most probable number (MPN) method in liquid phase. Indeed, when grown in liquid phase, bacteria can enter a viable but not culturable state (VBNC) and are not able to form colonies but still exhibit metabolic activity [39–41]. For the He-O_2_ plasma, after one hour, bacteria are still metabolically active but not culturable suggesting that this plasma may induce mild membrane damages resulting in the loss of culturability [29] (Fig 1D). However, two hours post-treatment following 10 min exposure to He plasma, were required to achieve 100% inactivation suggesting membrane disruption in *E*. *coli*. Flow cytometry analyzis confirmed these datas. Simultaneous staining with thiazole orange (TO) and propidium iodide (PI) allows the distinction of live (TO^+^PI^−^), dead (TO^+^PI^+^) with total membrane disruption, and injured (TO^+^PI^int^) cell populations, a viable but non-culturable VBNC form with no total membrane disruption [42]. The results showed that the population within the grey circle (TO^+^PI^−^) (Fig 1E), which refers to bacteria with high membrane potential disappeared regardless of the applied plasma treatment. The use of cell impermeable dye, propidium iodine, where fluorescence increases when the membranes are disrupted, indicated that treatment with He and He-N_2_ for 10 min with one hour post storage resulted in complete membrane disruption (TO^+^PI^+^), which is not the case for He-O_2_ plasma treatment which resulted in only injured cells with no membrane disruption but cell depolarization (TO^+^PI^int^), less staining for thiazole orange. Our results are in line with other studies [29, 42–44].

### Plasma treatment induced alteration in cell membrane

**Fig 2:**
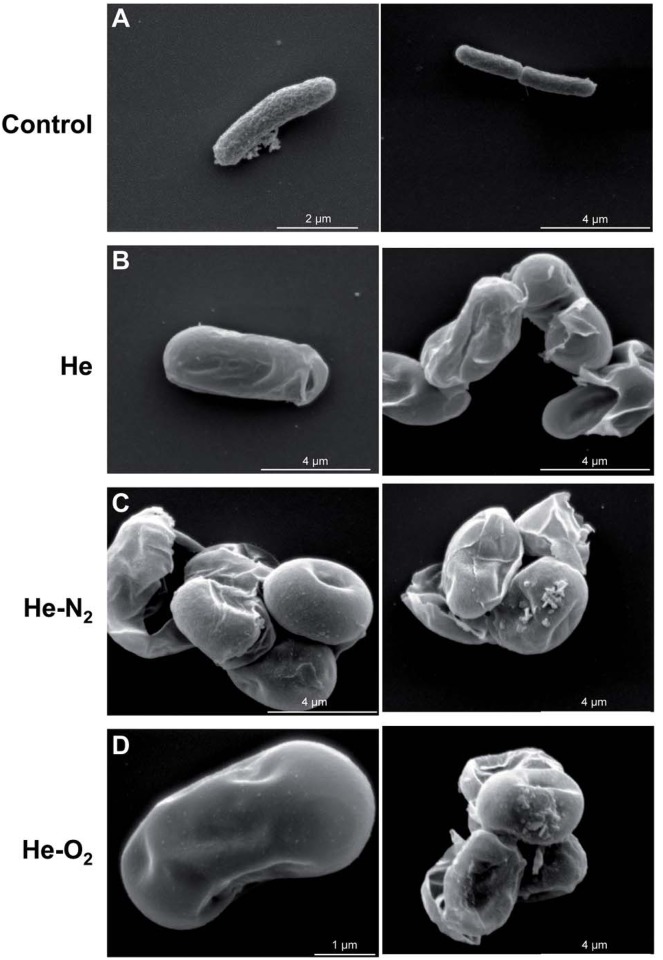
Effect of plasma exposure on bacterial morphology analyzed by SEM. A: Control (*E*. *coli*). Two pictures for each conditions are presented B: after 10 min He plasma treatment and 2 hour post-treatment storage at 4°C. C: after 10 min He-N_2_ plasma treatment and 2 hour post-treatment storage. D: after 10 min He-O_2_ plasma treatment and 2 hour post-treatment storage.

It was also interesting to focus on the morphological changes that may have occurred in *E*. *coli* post plasma treatment. SEM analysis indicated that 2 hour storage after 10 min of He, He-N_2_ and He-O_2_ plasma treatment, a condition resulting in 100% bacteria inactivation (Fig 1), caused the bacteria membranes to be completely disrupted (Fig 2B–2D). Formation of holes in the membrane were observed as well as morphological changes. Some bacteria were no longer rod-shaped but rounded, becoming coccoid and losing their bacillus shape. Such damage has been described in the literature [29, 42–44] and could be attributed to charged particles or electric field. To minimize the electrostatic forces exerted by charges, bacteria become round and if the charge effect or the electric field is too great, the membranes will break. By oxidation of the lipid membrane, RONS may also contribute to weaken bacterial membranes. In any case, it seems obvious that bacteria are permeabilized and loss of cellular components occur, confirming the results obtained by flow cytometry analysis (Fig 1E).

### Origin of the deleterious effect of plasma treatments on *E*. *coli*

**Fig 3:**
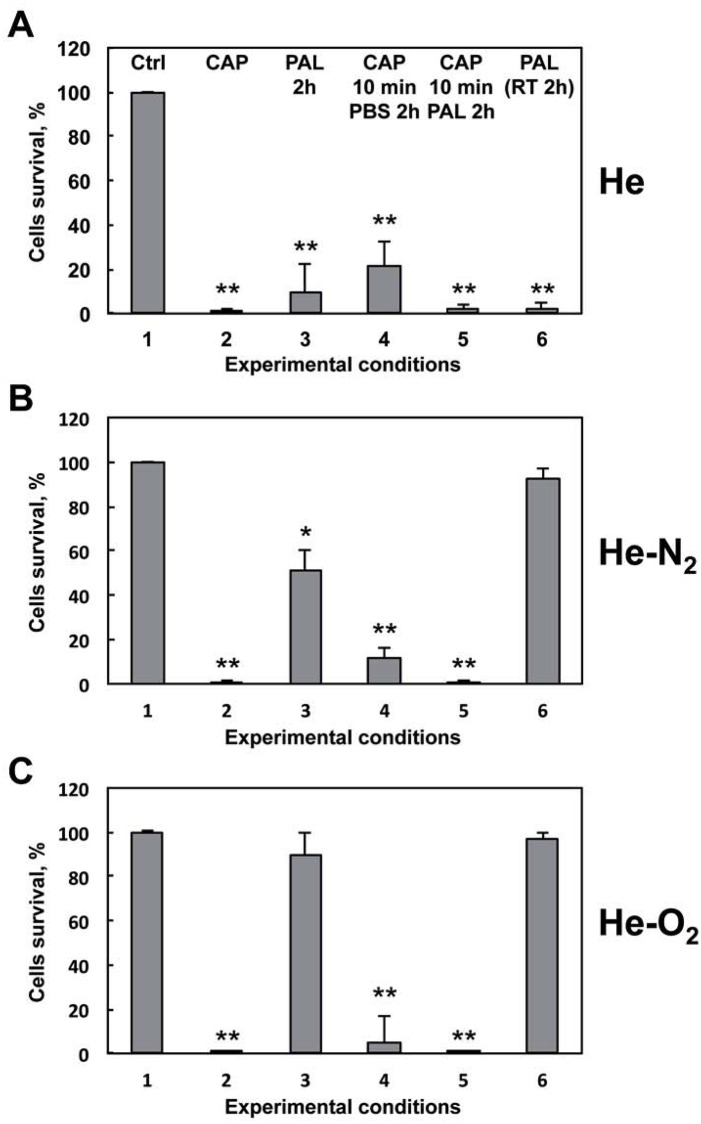
Effect of Plasma Activated Liquid (PAL) on bacteria viability. A: 1: control bacteria incubated for 2 hours in PBS and % of surviving cells was evaluated by the CFU method. 2: *E*. *coli* exposed to He plasma for 10 min with 2 hr storage at 4°C. 3: Bacteria exposed to PAL (PBS treated for 10 min He plasma) for 2 hr at 4°C. 4: Bacteria exposed to He plasma for 10 min and incubated for 2 hours with non-plasma treated PBS at 4°C. 5: Bacteria exposed to plasma for 10 min and then incubated for 2 hours at 4°C with PAL (PBS treated for 10 min He plasma). 6: Bacteria exposed to PAL (PBS treated for 10 min He plasma and left at room temperature for 2 hours) for 2 hours at 4°C. The values are means ± SEM of 3 separate experiments (*p<0.01 and ** p<0.05 vs control). B: Same experimental procedures using He-N_2_ plasma. C: Same experimental procedures using He-O_2_ plasma.

Because a post-treatment storage time is required to completely inactivate the bacteria after plasma exposure and in order to gain further insight into the mechanism by which plasma treatment kills bacteria, we compared the efficacy of direct treatment and Plasma Activated Liquid (PAL). We used PBS treated for 10 min with the three types of plasmas He, He-O_2_ and He-N_2_ and incubated for two hours with bacteria. For pure He plasma (Fig 3A), we found that PAL induced the same inactivation as direct plasma exposure suggesting that the species formed in the liquid medium after He plasma treatment played a central role in the potential mechanism of bacteria inactivation. However, for He-N_2_ plasma (Fig 3B), there was a significant difference between the effects obtained with PAL and with a direct 10 min plasma exposure, indicating that species produced in the liquid medium are not sufficient to inactivate bacteria. Thus transient species present in the plasma might react with cell components suggesting that energetic components contained in the plasma, are very important for achieving complete bacteria inactivation. For He-O_2_ plasma activated liquid (Fig 3C), no decline in cell viability was observed indicating that the He-O_2_ mechanism of action may involve UV, dissolved electrons, charged particles or others short lived species but not the RONS present in the liquid. Therefore the cell depolarization that is only observed with this plasma is likely to reflect the combined effects of charged particles and electric field. This plasma is definitely very different from the other two in its mode of action.

Despite no contact of the bacteria with the high voltage electrodes generating the plasma, an electrical connection exists. The propagation of the plasma channel transports an intense field and creates charged and neutral particles that induce a strong localized electric field [45–47]. If these factors contribute to bacterial inactivation, plasma treatment of the bacteria and incubation with non-treated PBS should kill them. The objective was to understand to what extent the energetic components contained only in the plasma phase are involved in the observed deleterious effect. Note that for the three plasmas (Fig 3A to 3C), most of the bacteria died as soon as they were submitted to the energetic components contained in plasma. It is now confirmed that not only the species produced in PBS but also energetic components present in the plasma, participate in the deleterious effects of the treatment. Additional controls were performed in order to check if treating PBS and bacteria separately, led to results similar to those obtained when they were treated together (Fig 3A to 3C). Our results suggested that in this condition there is a synergetic effect of the plasma energetic components and the RONS produced in the liquid. Taken together, our findings provide insight into potential mechanisms of the three plasma—induced bacteria inactivations. For pure He, bactericidal effects comes mainly from species formed in PBS. For He-N_2_, energetic components contained in the plasma and RONS present in the liquid are responsible for its bactericidal effects. It is likely that they act synergistically. Finally, for He-O_2_ plasma, what happens during the plasma exposure is totally responsible for the bactericidal effect by producing short-lived reactive species such as ^1^O_2_, O_2_^•-^, ^•^OH, etc. and we cannot rule out the electric field associated with the guided ionization wave or generated in the environment of the CAPP [29, 48, 49]. An interesting question was whether the species contained in the PAL are stable over time. Our experiments indicated that for pure He plasma, bactericidal properties of the PAL remained the same even 2 hours later. We did not observe the same with the He-N_2_ and He-O_2_ plasmas, after 2 hours the PAL is no longer toxic, suggesting that for these plasmas, short-lived species such as singlet oxygen, atomic oxygen or peroxynitrite may be involved in bacterial inactivation.

### Hydrogen peroxide and nitrites are the main RONS in PAL

**Fig 4:**
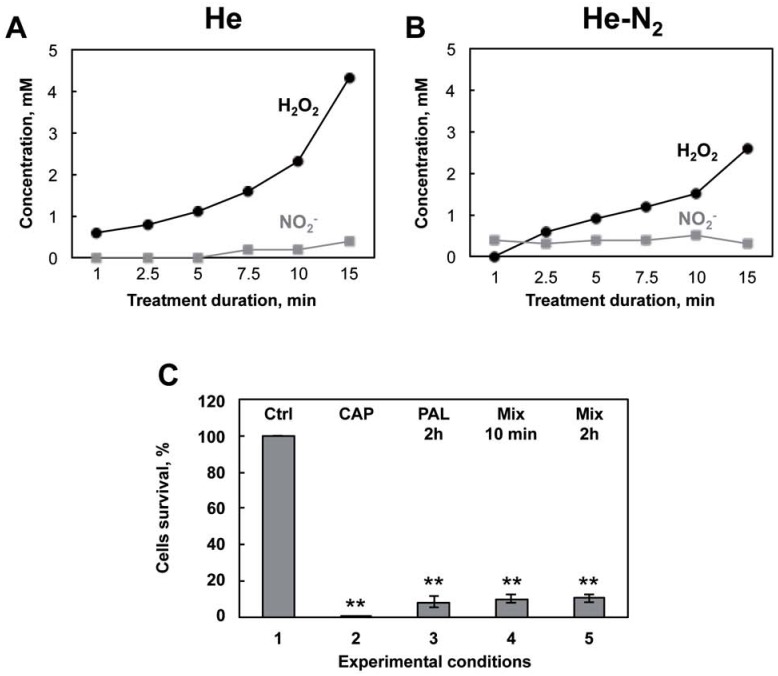
Nature, concentrations and toxicity of the species produced in PAL. A: Detection by cyclic voltammetry at a platinized microelectrode of hydrogen peroxide and nitrites produced in PBS after 1 min to 15 min of He plasma treatment. B: He-N_2_ treatment. C: Model experiment mimicking PAL. 1: control bacteria incubated for 2 hours in PBS. 2: *E*. *coli* exposed to He plasma for 10 min and 2 hr storage and % of surviving cells evaluated by the CFU method. 3: Bacteria exposed to PAL (PBS treated for 10 min, He plasma) for 2 hours for 2 hours at 4°C. 4: Bacteria exposed to a mix (2.3 mM Hydrogen peroxide and 0.5 mM nitrite) for 10 min or 5: for 2 hours for 2 hours at 4°C and % of surviving cells was evaluated by the CFU method. The values are means ± SEM of 3 separate experiments (** p<0.05) vs control).

CAPPs are known to generate RONS in PBS [48, 49]. It was therefore of interest to determine and quantify the species produced by the plasma in the liquid medium. To identify the species produced in PBS and their concentrations, PBS was treated as usual with plasma He and He-N_2_ for times ranging from one to 15 min. At the end of the treatment, treated samples were analyzed by cyclic voltammetry (Fig 4). Analysis indicated that two species are produced in the liquid medium, namely H_2_O_2_ and NO_2_^-^. As expected, Fig 4A and 4B, indicate that He and He-N_2_ plasmas are able to form these two species in PBS in a time dependent manner. H_2_O_2_ produced in the PBS is much higher with pure He plasma than with He-N_2_ plasma (2.3 mM and 1.5 mM, respectively, both at 10 min) whereas concentrations in NO_2_^-^ are roughly equivalent for the two plasmas (0.5 mM at 10 min). In the medium treated with He-O_2_, species considerably lower, being practically undetectable by cyclic voltammetry (detection limit of 200 μM) were measured. Therefore, absorption and fluorescence spectrophotometry were used to measure these species for He-O_2_ plasma treated solutions. We measured 40 μM H_2_O_2_ and 5 μM NO_2_^-^ at 10 min. H_2_O_2_ and NO_2_^-^ are species known to be chemically stable and potently toxic to cells depending on their concentration level, which could be responsible for the PAL effects. If these species contributed to bacterial inactivation in PBS we should be able to mimic plasma treatment. As shown in Fig 4C, H_2_O_2_ and NO_2_^-^ were added to non-plasma treated PBS at concentrations determined for 10 min of He plasma treatment and added to the bacteria for different conditions and compared to direct treatment with He plasma (Fig 4C). The same results are obtained when the two species are added together. Finally, with 10 min contact with bacteria, using this “mimicking medium”, the deleterious effect is already at a maximum, highlighting the toxicity of these two species and in line with what was observed in Fig 3B. Collectively, these findings suggest that H_2_O_2_ and NO_2_^-^ are generated in significant amounts and that they are the major RONS in He and He-N_2_ plasma treated PBS.

### Plasma treatment induced protein oxidation but no lipid peroxidation

**Fig 5:**
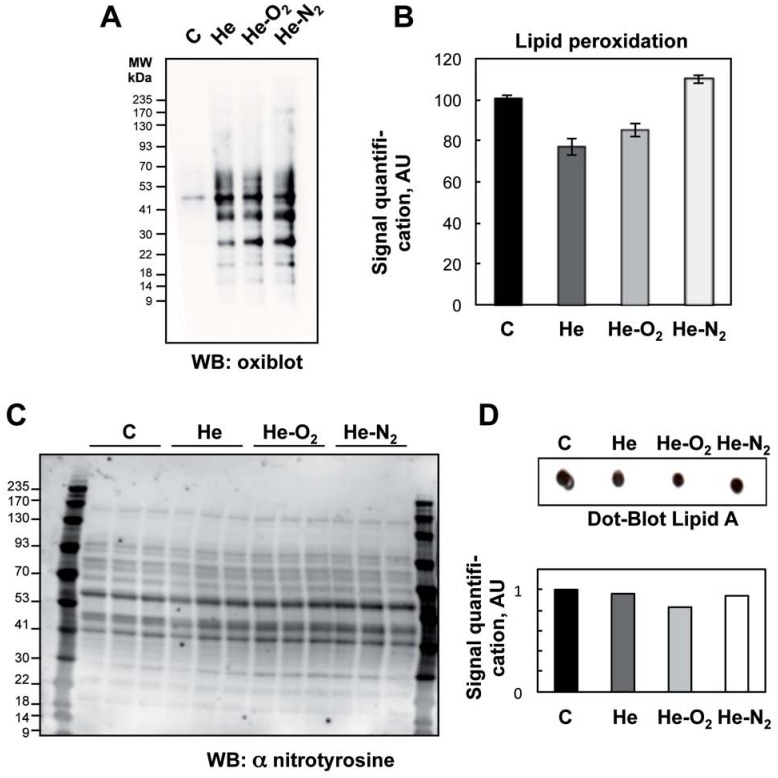
Detection of oxidatively modified proteins following plasma exposure. A: Bacteria exposed to plasma treatment (He, He-O_2_ and He-N_2_) for 10 min with 2 hours post-treatment storage. To detect oxidatively, modified protein bacterial extracts were treated with 2,4-dinitorphenylhydrazine to derivatize protein carbonyls and then evaluated by SDS-gel electrophoresis using 2,4-dinitrophenyl antibodies. B: Detection of 4-hydroxy-2-nonenal protein modification by ELISA. The values are means ± SEM of 3 separate experiments. C: Western blot analysis using nitrotyrosine specific antibodies. D: Bacterial extracts collected, lysed and detected by dot blot for lipid A content. Dot blot results were analyzed with a dot calibration curve and relative quantity of bacteria lipid A was estimated. The relative intensity of each spot was quantified (Image J).

Plasma treatment results in increased rates of RONS production in the PBS (Fig 4). It was therefore of interest to determine whether bacterial proteins were a target for oxidative modification. This was accomplished by evaluating changes in the relative levels of carbonyl groups present on the proteins. Carbonyl functional groups can be introduced into proteins by a variety of oxidative processes including direct oxidation of amino acid with H_2_O_2_ and reaction of lipid peroxidation products from cellular membrane oxidation [50]. As shown in Fig 5A, He, He-N_2_ and He-O_2_, plasma treatment induced a distinct increase in levels of oxidized proteins. Oxidative modifications were not global in nature but appeared specific to distinct low molecular weight proteins and no high molecular weight aggregates. Surprisingly, the level of protein oxidation was the same for all three plasmas suggesting that a low level of H_2_O_2_ or other species produced in the plasma by He-O_2_ such as ^•^O_2_ or ^1^O_2_ plasma were sufficient to induce protein oxidation. 4-Hydroxy-2-nonenal (HNE), an α,β unsaturated aldehyde is a major product of lipid peroxidation and very toxic [51]. Utilizing antibodies specific to HNE-Michael adducts we did not detect an increase in the HNE content of protein after plasma treatment compared to control cells (Fig 5B) suggesting that membrane rupture and changes in cell permeability are not due to peroxidation and oxidation of the membrane lipid and may be due to a depolarization of the cell [29]. While protein carbonylation is an irreversible modification due to H_2_O_2_ interaction with cell components, nitrotyrosine in protein is the detectable marker for indirectly detecting peroxynitrite ONOO^−^. It is a highly reactive species, since once formed, it decomposes in aqueous solution into highly reactive hydroxyl ^•^OH and nitroxyl NO_2_° [52]. Analysis of nitrated protein (Fig 5C) revealed no difference in nitrotyrosine amount on protein in plasma compared to control cells suggesting no formation of ONOO^−^ or its rapid degradation in the PBS during plasma treatment. It has recently been shown that *E*. *coli* cytochrome *bd* is able to catalyze the rapid degradation of ONOO^−^ [53]. So we cannot rule out that the ONOO^−^ degrading activity of cytochrome *bd* in the bacteria resulted in the formation of nitrite preventing tyrosine nitration.

Lipid A is an essential component of Gram-negative bacteria membrane. It serves as an anchor for lipopolysaccharides, LPS, that constitute the outer monolayer of the outer membrane. The ability of LPS to elicit an immune response lies essentially with lipid A [54]. It is a very potent stimulant of the immune system, which activates the cells (monocytes, macrophages) at concentrations of a few picograms per milliliter. At high concentration in the body in a gram-negative infection, it can trigger a systemic inflammatory response syndrome, which may cause sepsis [55]. In order to investigate if plasma treatment was able to degrade Lipid A, we used a dot blot method based on immuno-detection of Lipid A [38]. As shown in Fig 5D, no change in the amount of Lipid A was detected after plasma treatment and the results are the same for the three plasmas used. Since SEM and cell viability (Fig 2) results indicated a strong alteration in the *E*. *coli* membrane integrity and structure, we did not expect this result but it was in line with the lack of membrane lipid peroxidation.

Nevertheless, we observed severely damaged membranes, after plasma exposure, see Fig 2. The membrane is a prime cellular target during plasma treatment. RONS emitted from the plasma are believed to be a key factor in bacterial inactivation [8, 56], the results presented in this study confirm this hypothesis. Some authors have reported ROS induced loss of membrane integrity [18, 23, 29], but several stress factors occurred at the same time [57]. Free radicals and charged particles (electrons, ions) may act and cause surface lesions in membranes by direct bombardment [48, 49]. So, in our conditions we hypothesize that, rather than oxidative stress, charges or electric field induce significant membrane damage. Localized lesions in the membrane allowed further penetration of toxic reactive plasma species into the cell and facilitate the diffusion of RONS and other particles through the membrane, both causing severe damage to intracellular macromolecules [25]. Intracellular proteins are key regulators of bacterial function and are sensitive to oxidative stress [58]. Fig 4C shows that the three plasmas induced significant oxidative damage to intracellular proteins. This high level of protein oxidation participates in bacterial death by leading to dysregulation of cellular signalization [44]. This phenomenon might explain the delayed effect that was observed (Fig 1). It is also interesting to see that the He-O_2_ plasma, which does not produce stable RONS in liquid media, leads to the same level of oxidation as for the other plasmas. This could indicate that other oxidative species such as singlet oxygen are contained in the He-O_2_ plasma. On the contrary, the oxidative damage observed with He and He-N_2_ could be both attributed to significant concentrations of RONS produced in the liquid media. This is supported by studies indicating that bacteria suffer oxidative damage and protein oxidation during plasma treatment in liquid environments [25].

## Conclusion

In summary, our results provide support for the hypothesis that upon treatment with He and He-N_2_, free radicals generated in the PBS mediate bacterial inactivation thereby altering protein homeostasis and membrane integrity. However Lipid A is not degraded and remains toxic. Future studies are needed to identify plasma conditions to neutralize this cytotoxic toxin [38]. Their efficiency is dependent on treatment time and post-treatment storage duration because these parameters determine the concentration of reactive species essential for microbial inactivation. He-O_2_ plasma is the most efficient plasma for bacterial inactivation and seems to involve an electric field associated with the ionization front or, generated in the plasma environment. The inactivation of bacteria by electrochemical means has been well characterized [59]. It involves different mechanisms such as changes in the membrane potential which surely may lead to local ion flux imbalances. Death occurs due to either the formation of permanent pores and subsequent destabilization of the cell membrane, or loss of important cell components and destruction of chemical gradients via transport through transient pores [60]. If reactive RONS are present, these pores may allow the oxidants to freely access to the interior of the cell thus improving at a platinized microelectrode the inactivation process [44]. Electric fields are also capable of destroying cells without destroying their membranes. Matsunaga et al. described a system in which cells were killed without rupturing, but rather with the electrochemical oxidation of intracellular coenzyme A [61]. Ongoing efforts to identify reactive species produced in the liquid, after treatment with He and He-O_2_, and the impact of plasma generated electric field will enable the elucidation of the bacterial mechanism of inactivation.
